# Associations between Nature Exposure and Health: A Review of the Evidence

**DOI:** 10.3390/ijerph18094790

**Published:** 2021-04-30

**Authors:** Marcia P. Jimenez, Nicole V. DeVille, Elise G. Elliott, Jessica E. Schiff, Grete E. Wilt, Jaime E. Hart, Peter James

**Affiliations:** 1 Department of Epidemiology, Harvard T. H. Chan School of Public Health, Boston, MA 02215, USA; 2 Department of Population Medicine, Harvard Pilgrim Health Care Institute and Harvard Medical School, Boston, MA 02215, USA; pjames@hsph.harvard.edu; 3 Channing Division of Network Medicine, Department of Medicine, Brigham and Women’s Hospital and Harvard Medical School, Boston, MA 02215, USA; eelliott@hsph.harvard.edu (E.G.E.); rejch@channing.harvard.edu (J.E.H.); 4 Department of Environmental Health, Harvard T. H. Chan School of Public Health, Boston, MA 02215, USA; jschiff@hsph.harvard.edu (J.E.S.); gwilt@g.harvard.edu (G.E.W.)

**Keywords:** health benefits, mental health, nature, greenness, green space

## Abstract

There is extensive empirical literature on the association between exposure to nature and health. In this narrative review, we discuss the strength of evidence from recent (i.e., the last decade) experimental and observational studies on nature exposure and health, highlighting research on children and youth where possible. We found evidence for associations between nature exposure and improved cognitive function, brain activity, blood pressure, mental health, physical activity, and sleep. Results from experimental studies provide evidence of protective effects of exposure to natural environments on mental health outcomes and cognitive function. Cross-sectional observational studies provide evidence of positive associations between nature exposure and increased levels of physical activity and decreased risk of cardiovascular disease, and longitudinal observational studies are beginning to assess long-term effects of nature exposure on depression, anxiety, cognitive function, and chronic disease. Limitations of current knowledge include inconsistent measures of exposure to nature, the impacts of the type and quality of green space, and health effects of duration and frequency of exposure. Future directions include incorporation of more rigorous study designs, investigation of the underlying mechanisms of the association between green space and health, advancement of exposure assessment, and evaluation of sensitive periods in the early life-course.

## 1. Introduction

The “biophilia hypothesis” posits that humans have evolved with nature to have an affinity for nature [1]. Building on this concept, two major theories—Attention Restoration Theory and Stress Reduction Theory—have provided insight into the mechanisms through which spending time in nature might affect human health. Attention Restoration Theory (ART) posits that the mental fatigue associated with modern life is associated with a depleted capacity to direct attention [2]. According to this theory, spending time in natural environments enables people to overcome this mental fatigue and to restore the capacity to direct attention [3]. The Stress Reduction Theory (SRT) describes how spending time in nature might influence feelings or emotions by activating the parasympathetic nervous system to reduce stress and autonomic arousal because of people’s innate connection to the natural world [4,5]. Further, proponents of the biophilia hypothesis postulate that green spaces provide children with opportunities such as discovery, creativity, risk taking, mastery, and control, which positively influence different aspects of brain development [6]. Beyond the biophilia hypothesis, there are a number of other pathways through which nature may affect health, including but not limited to increasing opportunities for social engagement and space for physical activity, while mitigating harmful environmental exposures (e.g., air pollution, noise, heat) [7,8,9,10]. Though evidence is inconsistent, physical activity may serve as an important mechanistic pathway to beneficial health outcomes by providing increased opportunities for outdoor exercise (e.g., walking) and play [7,8,9]. Facilitation of social contact is a promising mechanism emerging from recent literature, where natural environments and green space provide an avenue for increased contact with others and a greater sense of community [9,10]. The mechanism’s underlying associations between nature exposure and health outcomes are many, not completely understood, and could act in isolation or synergistically [11].

While the study of exposure to nature and health outcomes has expanded substantially over recent years, there remain many understudied relationships, mechanisms, and populations. For instance, there is a much more expansive evidence base for associations between nature and health, particularly with experimental studies, in adults than in children. This narrative review synthesizes recent scientific literature on associations between nature and health, highlighting studies conducted among children and youth where possible, published throughout August 2020 and based on: (1) randomized experimental studies of short-term exposure to nature and acute responses; and (2) observational studies of exposure to nature.

## 2. Methods

A narrative review synthesizes the results of quantitative studies that employ diverse methodologies and/or theoretical frameworks without a focus on the statistical significance of the studies’ results [12,13]. We conducted a keyword search-based review using PubMed Advanced Search on 31 August 2020 for studies published in the last ten years with titles or abstracts containing “greenness”, “green space” or “NDVI” (i.e., normalized difference vegetation index) as the exposure, and “health, “children’s health” or “youth health” as the outcome (National Library of Medicine, Bethesda, MD, USA). Using World Health Organization definitions, we categorized a child as a person younger than 10 years and youth from 10 to 24 years inclusive [14]. We limited this narrative review to research on human subjects only and included English-language-based, international peer-reviewed articles (e.g., primary research, reviews), online reports, electronic books, and press releases. We included both experimental and observational studies and applied snowballing search methodology using the references cited in the articles identified in the literature search. Each identified item was assessed for relevance by a member of the study team. This review is not comprehensive but is intended to summarize recent literature on nature exposure and health.

## 3. Results

In retrieving literature on associations of nature and health, we reviewed a range of research from multiple health-related disciplines, geographic regions, and study populations. Evidence from the experimental and observational studies presented below represents more recent literature (e.g., the last decade) on nature exposure and health, primarily from Western countries.

### 3.1. Experimental Studies

We found a substantial body of research on natural environment interventions to evaluate the effects of nature on health from an experimental approach. The interventions consisted of active engagement in the natural environment (e.g., walking, running, or other activities), passive engagement (e.g., resting outside or living with a view), or virtual exposure (e.g., watching videos or viewing images of nature) [15,16]. The majority of experimental studies assessed mental health and neurologic outcomes. Results from experimental studies suggested a protective effect of exposure to natural environments on mental health outcomes and cognitive function.

#### 3.1.1. Stress

Several experimental studies have examined perceived stress and other subjective measures of stress, such as sleep quality. A recent systematic review of more than 40 experimental studies indicates that measures of heart rate, blood pressure, and perceived stress provide the most convincing evidence that exposure to nature or outdoor environments may reduce the negative effects of stress [17]. The results from perceived or reported stress after exposure to natural environments were more consistent than findings from studies using physiological stress measurements (e.g., cortisol levels) among adults. A recent meta-analysis found evidence suggesting that exposure to natural environments may reduce cortisol levels, one of the most frequently studied biological markers of stress. Song et al. [18] reviewed 52 articles from Japan that examined the physiological effects of nature therapy. There was overwhelming evidence that cortisol levels decreased when participants were exposed to a natural environment. In numerous studies, salivary cortisol levels decreased after mild to moderate exercise in a natural environment compared with an urban environment [18].

Although many studies have observed significant decreases in measured salivary cortisol levels after exposure to natural environments, others have not observed any significant differences in salivary cortisol levels before and after exposure to natural environments [17,19]. However, a key limitation of using cortisol as a biomarker of stress in experimental studies is the fluctuation of cortisol over a 24-h period. Diurnal cortisol levels need to be taken into account in order to make a fair comparison, and most of the literature on exposure to nature and stress have only studied cortisol levels before and after exposure [17].

Experimental studies focusing on children or youth are sparse [20,21]. One quasi-experimental study conducted in 10–12 year-olds in a school setting examined the influence of natural environments on stress response [22]. The researchers observed higher tonic vagal tone, a measure of heart rate variability, in natural environments but found no associations with event or phasic vagal tone.

#### 3.1.2. Affective State

Exposure to natural environments has also been studied in relation to the self-reported affective state, or the underlying experience of feeling, emotion or mood. Although study measures vary, studies among adults have generally observed relationships between exposure to natural environments and affective state, with positive associations with positive emotions and negative associations with negative emotions [16,22,23]. A study randomly assigned sixty adults to a 50-min walk in either a natural or an urban environment in Palo Alto, California, and found that compared to urban experience, nature experience led to affective benefits (decreased anxiety, rumination, and negative affect, and preservation of positive affect) as well as cognitive benefits (increased working memory performance) [23]. In a study investigating forest bathing, or shinrin-yoku, researchers found that time spent in forests was associated with a reduction in reported feelings of hostility, depression, and anxiety among adults with acute and chronic stress [24]. Another study examining walking in different environments observed the largest and most consistent improvements in psychological states associated with forest walks [25]. Forest bathing may play an important role in health promotion and disease prevention. However, the lack of studies focused on children or youth limits the generalizability of these findings across a wide age range [26].

#### 3.1.3. Anxiety and Depressive Mood

Exposure to natural environments has been linked with decreases in anxiety and rumination, which are associated with negative mental health outcomes, such as depression and anxiety [23,27]. Nature-based health interventions (NBI) are interventions that aim to engage people in nature-based experiences with the goal of improving health and wellness outcomes [28]. One study evaluated a wetland NBI in Gloucestershire, UK, that was designed to facilitate engagement with nature as a treatment for individuals diagnosed with anxiety and/or depression. The study found that the wetland site provided a sense of escape from participants’ everyday environments, facilitating relaxation and reductions in stress [27]. A recent systematic review and meta-analysis found a reduction in depressive mood following short-term exposure to natural environments [21]. However, the authors noted that the reviewed studies were generally of low quality due to a lack of blinding of study participants and a lack of information on randomization quality among randomized trials.

#### 3.1.4. Cognitive Function

Experimental studies have examined the impact of brief nature experiences and cognition among adults, investigating cognitive function related to exposure to natural environments, and are consistent with the results from studies among school-aged children. A growing number of studies have found that exposure to natural environments compared with urban environments is associated with improved attention, executive function, and perceived restorativeness [16,29,30,31,32,33,34,35,36,37]. These studies have found statistically significant associations with positive cognitive outcomes, even after short periods of time spent in natural environments. Additionally, an emerging area of research is virtual reality (VR), using eye-tracking and wearable biomonitoring sensors to measure short-term physiological and cognitive responses to different biophilic indoor environments. These studies have found consistent physiological and cognitive benefits in indoor environments with diverse biophilic design features [38,39].

#### 3.1.5. Brain Activity

Exposure to nature has been associated with alterations in brain activity in the prefrontal cortex, an area of the brain that plays an important role in emotional regulation [18,19]. One experimental study among female university students in Japan investigated physiological and psychological responses to looking at real plants compared with images of the same plants [40]. Although participants reported feelings of comfort and relaxation after seeing either real plants or images of the same plants, a physiological response was observed only after seeing real plants. Seeing real plants was associated with increased oxy-hemoglobin concentrations in the prefrontal cortex, suggesting that real plants may have physiological benefits for brain activity not replicated by images of plants.

#### 3.1.6. Blood Pressure

Two meta-analyses [18,41] found evidence suggesting that exposure to a natural environment reduced blood pressure. Song et al. [18] reviewed the research in Japan from 52 studies on the physiological effects of nature therapy and found overwhelming evidence that blood pressure levels decreased when participants were exposed to a natural environment. Decreases in both systolic and diastolic blood pressure levels were observed across young healthy populations, as well as populations with hypertension. This suggests that forest walking may lead to a state of physiological relaxation [18]. Ideno et al. [41] conducted another systematic review and meta-analysis to synthesize the effects of forest bathing on blood pressure, including 20 trials involving 732 participants including high-school and college-aged youth. The authors found that both systolic and diastolic blood pressure taken in the forest environment were significantly lower than in non-forest environments [41].

#### 3.1.7. Immune Function

In Japan, forest bathing has been positively associated with human immune function [42]. A study was conducted in which subjects experienced a 3-day/2-night bathing trip to forest areas, and blood and urine were sampled on days 2 and 3 of the trip. On days 7 and 30 after the trip, it was found that the mean values of natural killer (NK) cells (which play a major role in the immune system) and NK activity were higher on forest bathing days compared with control days [43]. This effect persisted for 30 days after the trip. A potential pathway for improved immune function is exposure to phytoncides (a substance emitted by plants and trees to protect themselves from harmful insects and germs), which could decrease stress hormones in the human body and increase NK cell activity. Additionally, the findings indicated that a day trip to a forest park also increased the levels of intracellular anti-cancer proteins [43].

#### 3.1.8. Postoperative Recovery

While there is limited research on the effect of nature on postoperative recovery, a seminal study by Ulrich [4] investigated recovery after a cholecystectomy on patients with and without a room with a window view of a natural setting. Patients with a view of a natural setting had shorter hospital stays, received fewer negative evaluative comments in the nurse’s notes section of their charts, and took fewer potent analgesics (e.g., opiates) than those patients whose windows faced a brick building wall [4]. More recent research has successfully replicated the concept that plants and foliage in the hospital environment may have beneficial impacts on surgical recovery in randomized trials [44,45].

### 3.2. Observational Studies

Cross-sectional observational studies have shown evidence of positive associations between exposure to nature, higher levels of physical activity, and lower levels of cardiovascular disease. Increasingly, longitudinal observational studies have started to examine the long-term effects of exposure to nature on depression, anxiety, cognitive function, and chronic disease. Below, we summarize the key findings on mental health, physical activity, obesity, sleep, cardiovascular disease, diabetes, cancer, mortality, birth outcomes, asthma and allergies, and immune function.

#### 3.2.1. Mental Health

A recent systematic review found limited evidence suggesting a beneficial association with mental well-being in children and depressive symptoms in adolescents and young adults [21]. However, access to green space has been linked with improved mental well-being, overall health, cognitive development in children [46], and lower psychological distress in teens [47]. A study that examined the restorative benefits associated with frequency of use of different types of green space among US-based students found that students who engaged with green spaces in active ways ≥15 min four or more times per week reported a higher quality of life, better overall mood, and lower perceived stress [48]. Research in the U.S.-based Growing Up Today Study (GUTS) found that increased exposure to greenness measured around the home was associated with a lower risk of high depressive symptoms cross-sectionally (as measured with the McKnight Risk Factor Survey) and a lower incidence of depression longitudinally [49]. The investigators observed stronger associations in more densely populated areas and among younger adolescents [49]. Similarly, a study in four European cities (Barcelona, Spain; Doetinchem, The Netherlands; Kaunas, Lithuania; and Stoke-on-Trent, UK) that evaluated childhood nature exposure and mental health in adulthood showed that adults with low levels of childhood nature exposure had, when compared with adults with high levels of childhood nature exposure, significantly worse mental health, assessed through self-reports of nervousness or depression [50]. Another study of approximately one million Danes over 28 years of follow-up found that high levels of continuous green space presence during childhood were associated with lower risk of a wide spectrum of psychiatric disorders later in life [51]. A study based in the UK tracked individuals’ residential trajectories for five consecutive years and showed that individuals who moved to greener areas had better mental health than before moving [52]. Collectively, these studies suggest that implementation of environmental policies to increase urban green space may have sustainable public health benefits.

Novel research has examined green outdoor settings as potential treatment for mental and behavioral disorders, such as attention-deficit/hyperactivity disorder (ADHD). One study demonstrated associations between green space exposure and improvement in behaviors and symptoms of ADHD and higher standardized test scores [46]. A recent systematic review found significant evidence for an inverse relationship between green space exposure and emotional and behavioral problems in children and adolescents [21]. Research has also shown that more and better quality residential green spaces are favorable for children’s well-being [53] and health-related quality of life [54]. Furthermore, the quality of green space appears to be more important as children age, as associations between green space quality and well-being are stronger in 12–13 year-olds compared with 4–5 year-olds [53]. In addition, natural features near schools, including forests, grasslands, and tree canopies, are associated with early childhood development, preschoolers’ improvement in socio-emotional competencies [55], and a decrease in autism prevalence [56].

Exposure to nature during adulthood also appears to be important for mental health. A study of 94,879 UK adults indicated a consistent protective effect of greenness on depression risk that was more pronounced among women, participants younger than 60 years, and participants residing in areas with low neighborhood socioeconomic status or high urbanicity [57]. Other innovative studies are starting to examine quantifiable time of exposure to evaluate the duration of time spent in nature that is associated with mental health benefits. For example, using a nationally representative sample of American adults, Beyer et al. [58] found that individuals who spent 5–6 or 6–8 h outdoors during weekends had lower odds of being at least mildly depressed, compared with individuals who spent less than 30 min outdoors on weekends. Another study from the UK suggested that lower levels of depression were associated with spending five hours or more weekly in a private garden [59]. Other studies are focused on uncovering which characteristics of green space are the determinants of mental health benefits. A UK study examined neighborhood bird abundance during the day and found inverse associations with prevalence of depression, anxiety, and stress [60].

The collective results from these studies suggest that nearby nature is associated with quantifiable mental health benefits, with the potential for lowering the physical and financial costs related to poor mental health. Most of these studies are cross-sectional, and reverse causation is possible. However, researchers are employing novel designs to examine the relationship between green space and mental health. For example, in a study of twins enrolled in the University of Washington Twin Registry, increased greenness was associated with decreased risk of self-reported depression, stress, or anxiety; however, only the results for depression were robust in within-twin pair analyses, suggesting the effect of green space on depression cannot be explained by genetics alone [61]. Finally, it is important to note that technological advancements have yielded improvements in assessments of exposure to nature and mental health. For instance, one study among adults 18–75 years of age used smartphones equipped with ecological momentary assessment applications to track location, physical activity, and mood for consecutive days, and found positive associations with feeling happy and restored or relaxed within 10 min of exposure to natural outdoor environments [62]. More novel studies such as these will bolster the evidence behind exposure to nature and mental health among children and/or youth.

#### 3.2.2. Physical Activity

An extensive body of literature documents the impacts of access to green spaces or surrounding greenness on physical activity in children and adults. Proximity to green spaces may promote physical activity by providing a space for walking, running, cycling, and other activities. Although the bulk of the literature is cross-sectional, most studies (in both children and adults) have observed higher levels of physical activity in areas with more access to green space. For example, a study in Bristol, UK, evaluated associations between accessibility to green space and the odds of respondents achieving a recommended 30 min or more of moderate activity five times a week; respondents who lived closest to the type of green space classified as a formal park were more likely to achieve the physical activity recommendation [63]. Another study of adults in the UK found that people living in greenest compared with least-green areas were more likely to meet recommended daily physical activity guidelines [64]. However, another UK-based study did not find associations between road distance to nearest green space, number of green spaces, area of green space within a 2-km radius of residence, or green space quality and physical activity [65].

Almanza et al. [66] used GPS and accelerometry data among 208 children in California and found that greenness was associated with higher odds of moderate to vigorous physical activity, when comparing those in the 90th and 10th percentiles of greenness. Additionally, they found that children with >20 min daily green space exposure had nearly 5 times the daily rate of moderate to vigorous physical activity compared with those with nearly zero daily exposure [66]. Another study of Australian children illustrated that time spent outdoors at baseline positively predicted the amount of physical activity three years later [67]. In a review of youth health outcomes related to exercising in nature (i.e., “green exercise”), the results of fourteen studies (5 in the UK, 5 in the U.S., 2 in Australia, and 1 in Japan) indicated little evidence that green exercise is more beneficial than physical activity conducted in other locations, although any physical activity was beneficial across settings [68].

More recent studies have employed more sophisticated study designs to determine whether exposure to greenness increases physical activity. In studies that objectively assessed physical activity via accelerometers, individuals exposed to more greenness tended to be more physically active. For example, in a study of 15-year-olds in Germany, increases in greenness around the home address were associated with increased moderate-to-vigorous physical activity among youth in rural, but not urban, areas [69]. Another study of children in the UK evaluated momentary green space exposure based on GPS-derived location and contemporaneous physical activity measured by an accelerometer and found higher odds of physical activity in green space (versus outdoor non-green space) for boys but not girls [70].

#### 3.2.3. Obesity

Green space may influence overweight or obesity through a physical activity pathway [71]. Some studies have shown that exposure to green space is associated with lower rates of obesity in children [67] and adults [72]; however, the results are conflicting. As with physical activity, many early studies were cross-sectional, and findings were more mixed for children than for adults. Some studies reported U-shaped associations with obesity [73], while other studies reported no association after adjustment for respondent characteristics [63] or neighborhood socioeconomic status [74]. Some studies demonstrate effect modification by gender [72]. Further, one cross-sectional UK-based study found that living in the greenest areas was associated with an increase in risk of being overweight and obese [75].

In one study of U.S. children, increasing greenness was associated with lower BMI z-scores and lower odds of increasing BMI z-scores between two follow-up times [76]. Another study of schoolchildren in Spain found that greenness and forest proximity were associated with lower prevalence of being overweight or obese [77]. One study found that street tree density was associated with lower obesity prevalence in New York City (U.S.) children; however, no association was found with park areas [78]. In an Australian study, the prevalence of being overweight was 27–41% lower in girls and boys who spent more time outdoors at the study baseline than those who spent less time outdoors [67]. Another study found that greenness was associated with decreased risk of being overweight but only among those in areas with a greater population density [79].

#### 3.2.4. Sleep

Exposure to green space may influence sleep duration and quality. For instance, surrounding greenness may serve as a buffer for noise, which would disturb sleep. To date, only a handful of studies have examined these associations, and to our knowledge, even fewer have explored this association in children. A recent systematic review provided evidence of an association between green space exposure and improved sleep quality among adults [80]. A study of Australian adults who lived in areas with greater than 80% green space demonstrated lower risk of short sleep duration, even after adjustment for other predictors of sleep [81]. Among U.S. adults participating in the Behavioral Risk Factor Surveillance System survey, natural amenities (e.g., green space, lakes, and oceans) were associated with lower reporting of insufficient sleep, and greenness was especially protective among men and individuals over 65 years of age [82]. In the Survey of Health in Wisconsin Study, increased tree canopy at the Census block group level was associated with lower odds of short sleep duration on weekdays and suggestive of an association with lower odds of short sleep duration on weekends, although there was no association between tree canopy and self-reported sleep quality [83]. A nationally representative study of Australian and German children and adolescents found no evidence of significant associations between residential green space and insufficient sleep or poor sleep quality [84].

#### 3.2.5. Cardiovascular Disease

Exposure to green space may affect levels of physical activity, stress, and high blood pressure that drive cardiovascular disease risk. Recent reviews have found consistent evidence that exposure to residential green space is associated with decreased cardiovascular disease incidence [85,86]. Participants living in areas with lower greenness have higher levels of mortality following a stroke [87], higher cardiovascular disease mortality [88,89], and higher coronary heart disease [90]. A study from the UK found that associations between exposure to nature and cardiovascular outcomes differed by gender, where male cardiovascular disease and respiratory disease mortality rates decreased with increasing green space, and no associations were found for women [88]. Furthermore, the relationships between exposure to greenspace and cardiovascular outcomes may be modified by urbanicity. A recent Australian study showed significantly lower odds of high blood pressure among adults in an urban population when reported green space visits were an average of 30 min or more [91].

#### 3.2.6. Diabetes

Although limited, evidence regarding the association between green space and type 2 diabetes highlights green space as a possible route for diabetes prevention. There are a few cross-sectional studies that have reported that green space is inversely related to type 2 diabetes among adults [92,93]. Few studies have examined the relationship between green space and diabetes in children. Cross-sectional studies of children found inverse associations between time spent in green spaces and fasting blood glucose levels [77] and insulin resistance [94]. A recent longitudinal study conducted on US children found no associations between residential exposure to green space and insulin resistance [95].

#### 3.2.7. Cancer

Research on the link between green space and cancer is limited and may vary depending on the type of cancer. A recent case-control study examined whether residential green space exposure was related to prostate cancer incidence and found that higher residential greenness was associated with lower risk of prostate cancer [96], and a separate study of U.S. men demonstrated inverse associations between neighborhood greenness and lethal prostate cancer [97]. Another study examined the association between green space and several cancer types and found that green space was protective for mouth, throat, and non-melanoma skin cancers but was not associated with colorectal cancer [98]. A U.S.-based nationwide study of nurses found that residential greenness was inversely associated with breast cancer mortality [99]. Conversely, another systematic review that evaluated evidence on the association between residential green spaces and lung cancer mortality found no benefits of residential greenness [85].

#### 3.2.8. Mortality

Many early mortality studies relied on cross-sectional data and could not estimate nature exposure over time [100], whereas others could not account for important potential confounding by race/ethnicity, individual-level smoking, and area-level socioeconomic factors, such as median home value [101,102]. A UK-wide ecological study found that all-cause mortality was higher in greener cities [89]. An analysis of greenness and mortality in male and female stroke survivors living in Boston (U.S.) found that greater exposure to greenness was associated with higher survival rates [87]. Another U.S.-based nationwide study of nurses found a consistent protective relationship between residential greenness and non-accidental mortality [103]. The greenness–mortality relationship was explained primarily by a mental health pathway, and the relationship was strongest among those who had high levels of physical activity [103]. A study of 4.2 million adults in the Swiss National Cohort assessed the relationship between residential greenness and mortality, while mutually considering socioeconomic status, air pollution, and transportation noise exposure, and found that higher exposure to green space was associated with lower rates of death from natural causes, respiratory disease, and cardiovascular disease [104]. Protective effects were stronger in younger individuals and in women and, for most outcomes, in urban (versus rural) and in the highest (versus lowest) socioeconomic quartile. Effect estimates did not change after adjustment for air pollution and transportation noise, suggesting that the protective effect of exposure to nature persists in the absence of pollution sources. Finally, a systematic review and meta-analysis of cohort studies on green space and mortality assessed findings from nine studies, comprising 8.3 million individuals from seven countries across the globe [105]. Seven of the nine studies demonstrated an inverse relationship between green space exposure and mortality, and the authors recommended wide-scale interventions to increase and manage green spaces in order to improve public health outcomes.

#### 3.2.9. Birth Outcomes

The relationship between exposure to nature and birth outcomes has been studied extensively in analyses across multiple countries. Findings of positive associations between greenness and birth weight and decreased risk of low birth weight are consistent, with stronger associations observed among those of a lower socioeconomic status [106]. Banay et al. [107] reviewed studies that examined the association between greenness and maternal or infant health. While the majority of studies were cross-sectional, many studies found evidence for positive associations between greenness and birth weight. Fewer studies demonstrated consistent evidence for an association between greenness and gestational age, preeclampsia, or gestational diabetes. These studies also found that effects were stronger among those of a lower socioeconomic status. A more recent review highlighted the evolving literature showing that higher levels of residential greenness were associated with lower risk of preterm birth, low birth weight, and small gestational-age babies [108]. Akaraci et al. [109] conducted a systematic review and meta-analysis of 37 studies on residential green and blue spaces and pregnancy outcomes. Increases in residential greenness were associated with higher birthweight and lower odds of being small for gestational age; however, no significant associations between residential blue spaces and birth outcomes were found.

#### 3.2.10. Asthma/Allergies

Several studies have examined the relationship between greenspace and atopic outcomes, including asthma and allergies. Mechanistically, trees and plants are a source of allergens and respiratory irritants [110]. However, the biodiversity created by green space could be protective against inflammatory conditions [111,112]. The literature reflects these contrasting hypotheses with mixed findings. Some studies have shown no association between the normalized difference vegetation index (NDVI) or tree canopy cover and asthma [113], while other studies have shown that living close to forests and parks was positively associated with allergic rhinoconjunctivitis and asthma [77]. Another study of greenspace and allergies in Germany demonstrated positive associations in urban areas and negative associations in rural areas [114]. The same investigators examined data from seven birth cohorts across Sweden, Australia, the Netherlands, Canada, and Germany and found that the relationship between residential NDVI and allergic disease was positive in some countries and negative in others [115]. A study in Spain found proximity to residential greenness to be protective of bronchitis in the Mediterranean region of Spain and protective of wheezing for children in the Euro-Siberian region of Spain [116]. One study conducted in China examined the relationship between exposure to greenness and parks and asthma and allergies among middle-school-aged children [117]. The researchers observed no associations between residential greenness exposure and self-reported doctor-diagnosed asthma, pneumonia, rhinitis, and eczema; however, living farther away from a park was associated with decreased odds of currently or ever having asthma. In sum, the relationship between exposure to nature and asthma and allergies is inconsistent, with associations varying in magnitude and direction by geography. One review of fourteen studies suggested an association between early life exposure to urban greenness and allergic respiratory diseases (e.g., asthma, bronchitis, allergic symptoms) in childhood; however, there were inconsistencies among study results, likely due to variability in study design, exposure assessment, outcome ascertainment, and geographic region [118].

### 3.3. Natural Experiments/Randomized Controlled Trials of Chronic Outcomes

Beyond smaller experimental studies of short-term outcomes and observational epidemiologic studies of chronic outcomes, there are a few natural experiments and randomized controlled trials that add substantial evidence to the relationship between exposure to nature and health. These quasi-experimental and randomized trials have lower potential for confounding bias to explain observed associations between nature and health. One important study capitalized on a natural experiment when an invasive tree pest, the emerald ash borer, killed over 100 million ash trees in the Midwestern United States [119]. The investigators found that living in a county infested with the emerald ash borer was associated with a 41% increased risk of cardiovascular disease, and these results were only consistent when looking in metropolitan areas where they could adjust for socioeconomic status. Another innovative study examined the greening of vacant lots in Philadelphia [120]. This citywide study used a three-arm randomized trial approach to randomize 110 vacant lots to either no intervention, cleaning but no greening, or cleaning and greening. The study found that those living around lots that were greened had substantial decreases in reports of depression, poor mental health, and feelings of worthlessness compared with lots that had no intervention. Those living around lots that were cleaned but not greened showed no difference compared with no intervention. Another ongoing longitudinal study in Sydney, Australia, is evaluating the effects of large-scale investment in green space (e.g., public access points, advertising billboards, walking and cycle tracks, BBQ stations, and children’s playgrounds) on physical activity, mental health, and cardiometabolic outcomes [121]. This natural experiment utilizes proximity to different areas of the Western Sydney Parklands to define treatment and control groups.

Looking to the future, there are a few randomized trials in progress that will provide fundamental evidence to understand whether adding green pace to cities benefits health. The Green Heart Project in Louisville, Kentucky, will assess risk of diabetes and heart disease, stress levels, and the strength of social ties in 700 participants [122]. The team will take baseline measurements of air pollution levels and will plant as many as 8000 trees, plants, and shrubs throughout Louisville neighborhoods to create an urban ecosystem that promotes physical activity while simultaneously decreasing noise, stress, and air pollution. During five years of follow-up, participants will receive annual check-ups to evaluate how the increasing greenery has affected their physical and mental health and social ties. A second randomized trial is the ‘Productive Green Infrastructure for Post-industrial Urban Regeneration’ or ProGIreg, a multi-city study examining the potential effects of green infrastructure [123]. This project is based in Dortmund (Germany), Turin (Italy), Zagreb (Croatia) and Ningbo (China) where Living Labs are hosted and nature-based solutions are developed, tested, and implemented. Although health is not the main focus of this study, researchers are hoping to incorporate health metrics into the study design to examine pre- and post-intervention outcome data. Collectively, these randomized trials, natural experiments, and pre-post study designs will establish crucial data on whether interventions to incorporate nature into cities can measurably improve health.

### 3.4. Effect Modification/Susceptible Populations

Inequitable distribution of green spaces could exacerbate health inequalities if people who are already at greater health risks (e.g., people with lower socioeconomic status) have limited access. Many studies have indicated that disadvantaged populations have decreased access to nature and greenspace [124,125,126,127,128,129,130,131,132]. At the same time, evidence suggests that exposure to nature disproportionately benefits disadvantaged populations, a phenomenon known as the equigenic effect of green space, which upends the expected association between lower socioeconomic status and greater risk of poor health outcomes [133]. Based on the theory of equigenic environments, one study showed that populations exposed to the greenest environments also had the lowest levels of health inequality related to income deprivation, suggesting that green space might be an important factor in reducing socioeconomic health disparities [89]. A review of 90 studies on green space and health outcomes demonstrated that individuals of lower socioeconomic status showed more beneficial effects than those of higher socioeconomic status; the authors found no significant differences in the protective effects of green space on health outcomes among different racial/ethnic groups [134]. The evidence is inconsistent, and more work is needed to elucidate potential mechanisms.

Conversely, improvements in access to green space may lead to “green gentrification,” an increase in property values that displaces low-income residents from their neighborhoods [129,135,136,137]. This process needs to be studied and understood so that its adverse effects can be prevented. Other cultural and contextual factors may affect nature preferences and experience of nature. For instance, there is evidence that the legacy of forced labor, lynching, and other violence may evoke deeply disturbing associations with trees, fields, and forests among some African Americans [138,139]. Similarly, some people may prefer open fields for sports, while others prefer picnic facilities for socializing.

## 4. Discussion

The purpose of this narrative review was to summarize recent experimental and observational literature on associations between nature exposure and health in adults and children/youth. While some associations between nature and health outcomes are well-studied, our review highlights the lack of studies, particularly experimental, among child/youth and other susceptible populations. We found evidence for associations between exposure to nature and improved cognitive function, brain activity, blood pressure, mental health, physical activity, and sleep. Results from experimental studies indicated protective effects of nature exposure on mental health and cognitive function. Cross-sectional observational studies provide evidence of positive associations between nature exposure, higher levels of physical activity and lower levels of cardiovascular disease. Observational studies, natural experiments, and randomized controlled trials are starting to assess the longitudinal effects of exposure to nature on depression, anxiety, cognitive function, chronic disease, and other health outcomes. Our review synthesizes recent literature, primarily from Western countries; thus, a limitation of this review is that we may not have captured all relevant literature from outside our publication range or across all geographic regions.

### 4.1. Data Gaps and Limitations

There are several limitations in the literature on exposure to nature and health. First, definitions of nature are inconsistent across studies. Further, the impacts of the quality of green space, duration of exposure to nature, frequency of exposure, or type of nature exposure on health outcomes are not well understood. Second, methods for measuring exposure to nature (e.g., percentage of residential greenness versus distance to the closest park) or defining the relevant geographic area of exposure (e.g., 500 m away from our home versus 1 km or 10 km) are inconsistent [140,141]. We must also develop methods to elucidate thresholds for dose and duration of nature exposure to achieve a given health effect. Although some studies have determined potential estimates of relevant doses [142], this area of research is nascent. In addition, standard approaches towards nature exposure assessment do not capture the variations in how people experience nature differentially (e.g., smell, touch, etc.) and have low reproducibility across studies (e.g., inconsistent land-use measures). Third, critical time windows of exposure during the life course that might have the greatest impact on health are also understudied (e.g., early life exposure, childhood exposure). Fourth, mechanistic pathways are understudied. Further, the dynamic relationship between green space, air pollution, noise, temperature, and neighborhood walkability also warrant further exploration, as these factors could be both mediators or moderators of the nature–health relationship [143,144]. We also know little about the potential harms of exposure to nature, most commonly observed in studies of asthma and allergies.

### 4.2. Future Directions

There are ample promising future directions for nature and health research. First, future research should employ rigorous study designs (e.g., longitudinal studies, randomized controlled trials) and investigate the underlying mechanisms of observed associations between exposure to nature and health outcomes. Although cross-sectional studies dominate the literature, there is increasing evidence emerging from prospective studies, which are essential to investigating causal relationships [108]. Novel designs, such as quasi-experimental studies and randomized trials, will provide further detail on how nature influences health [119]. Furthermore, studies should thoroughly evaluate potential biases, such as confounding by socioeconomic status, that may threaten the validity of studies on nature and health. Researchers should rigorously examine factors that may modify the effects of exposure to nature (e.g., socioeconomic status, gender, or race) to determine the subpopulations that might benefit most from exposure to nature. A life-course approach to examining associations between green space and health is also essential. We need to better understand vulnerable time windows in the early life-course where access or exposure to nature may have stronger impacts on health than in other time periods. Similarly, additional research assessing dose-response relationships (e.g., duration of time in nature or quantity of vegetation) is crucial to determine the minimum amount of exposure to green space needed to yield health benefits or if the relevant dosage varies across the life-course or across different countries/settings [142].

Second, future studies should make use of novel datasets and computational approaches that may provide rapid advances in exposure assessment. Emergence of advanced satellite and aerial photos combined with machine learning to develop tree canopy measures and other more specific metrics of nature provide information on specific species on the ground. Google Street View and other ubiquitous geocoded imagery, when combined with machine learning, also provide scalable approaches to estimate specific natural features from the on the ground perspective as human beings experience them [145]. Combined with geocoded residential addresses or GPS data and health or behavioral data, these approaches may unveil novel insights on how nature exposure affects health. Leveraging smartphones with GPS and accelerometry enable fine-scale information on exposure and physical activity. Ecological momentary assessment (EMA) or micro-surveys administered through smartphones can be used to ask about processes for how and why people interact with nature [62]. EMA can also be applied to estimate mental health outcomes in real time, and these responses can be geo-tagged and linked to spatial measures of natural environments. In addition, consumer wearable devices (e.g., FitBit) provide objective information on physical activity patterns, heart rate, sleep, and other biometrics down to the second level [146]. These data will prove crucial to better understand the behavioral mechanisms through which nature exposure impacts health. We should also capitalize on geo-located social media data (Flickr, Twitter, Facebook) and other data sources to understand exposure to nature [147]. Innovative metrics of mental health, such as skin conductivity, cortisol (stress), heart rate variability, brain activity through EEG, and functional MRI, can also provide information on stress processes when individuals encounter natural environments [148]. Such measures of nature exposure and time spent in nature should be incorporated into large federal data collection efforts, such as the Behavioral Risk Factor Surveillance System (BRFSS), National Health Interview Survey (NHIS), and National Health and Nutrition Examination Survey (NHANES) in the United States or the Health Survey for England (HSE) in the United Kingdom. These recommendations cannot be accomplished without also considering the impacts climate change is currently having and will have on exposures to nature, and how climate change may alter the relationship individuals have with nature.

Third, future studies on nature and mental health should focus more on positive health—happiness, purpose, flourishing—instead of just the absence of negative mental health outcomes. Further, more research is required on natural water features, or blue space [149], as well as other natural environments.

Fourth, the overwhelming majority of research on nature and health is on urban study populations in North America, Europe, and Australia. Researchers should also focus on different geographic areas, low-income and middle-income settings, and vulnerable or historically marginalized populations where nature benefits might be greatest. Researchers should also work together with communities as they conduct their research to ensure their work addresses the needs of community members.

Finally, we must also recognize the potential unintended consequences of adding green infrastructure in cities. Adding green amenities to cities may entice high-income populations, and the resulting increased property values shape a new conundrum, embodied in the exclusion and displacement associated with so-called green gentrification [135]. Results from this type of research should also be considered for policies, urban planning, and designing cities.

## 5. Conclusions

The purpose of this review was to examine recent literature on exposure to nature and health, highlighting studies on children and youth where possible. We assessed the strength of evidence from experimental and observational studies and found evidence for associations between exposure to nature and improved cognitive function, brain activity, blood pressure, mental health, physical activity, and sleep. Evidence from experimental studies suggested protective effects of exposure to natural environments on mental health outcomes and cognitive function. Cross-sectional observational studies provide evidence of positive associations between exposure to nature, higher levels of physical activity and lower levels of cardiovascular disease. Longitudinal observational studies are starting to assess the long-term effects of exposure to nature on depression, anxiety, cognitive function, and chronic disease. Limitations and gaps in studies of nature exposure and health include inconsistent measures of exposure to nature, knowledge of the impacts of the type and quality of green space, and the health effects of the duration and frequency of exposure among different populations (e.g., adults, children, historically marginalized). Future research should incorporate more rigorous study designs, investigate the underlying mechanisms of the association between green space and health, advance exposure assessment, and evaluate sensitive periods throughout the life-course.
